# Frequency of physical activity and stress levels among Brazilian adults during social distancing due to the coronavirus (COVID-19): cross-sectional study

**DOI:** 10.1590/1516-3180.2020.0706.R1.0802021

**Published:** 2021-06-25

**Authors:** Edina Maria de Camargo, Thiago Silva Piola, Letícia Pechnicki dos Santos, Edilson Fernando de Borba, Wagner de Campos, Sergio Gregorio da Silva

**Affiliations:** I PhD. Physical Education Teacher, Postgraduate Program on Physical Education, Universidade Federal do Paraná (UFPR), Curitiba (PR), Brazil.; II PhD. Physical Education Teacher, Postgraduate Program on Physical Education, Universidade Federal do Paraná (UFPR), Curitiba (PR), Brazil.; III MSc. Physical Education Teacher, Postgraduate Program on Physical Education, Universidade Tecnológica Federal do Paraná (UTFPR), Curitiba (PR), Brazil.; IV PhD. Physical Education Teacher, Postgraduate Program on Physical Education, Universidade Federal do Paraná (UFPR), Curitiba (PR), Brazil.; V PhD. Physical Education Teacher, Postgraduate Program on Physical Education, Universidade Federal do Paraná (UFPR), Curitiba (PR), Brazil.; VI PhD. Physical Education Teacher, Postgraduate Program on Physical Education, Universidade Federal do Paraná (UFPR), Curitiba (PR), Brazil.

**Keywords:** Epidemiology, Pandemics, Exercise, SARS, Stress, Physical activity

## Abstract

**BACKGROUND:**

The COVID-19 pandemic may be having many psychological impacts on people, at both an individual and a community level.

**OBJECTIVE:**

To ascertain the relationship between the weekly frequency of physical activity and levels of stress among Brazilian adults during social distancing due to the coronavirus (COVID-19), and the interaction of sex in this association.

**DESIGN AND SETTING:**

Cross-sectional study with a descriptive approach conducted at a public university in Curitiba (PR), Brazil.

**METHODS:**

2,000 Brazilian adults (average age 36.4 years; 59.6% women) were recruited according to convenience through digital media. They filled out a questionnaire in electronic format that asked for sociodemographic information, health data, food consumption data, weekly frequency of physical activity and stress levels on the 10-item Kessler psychological distress scale. Descriptive statistics and regression analyses were used to evaluate the data.

**RESULTS:**

Associations were observed for the following correlations: male sex * no physical activity (odds ratio (OR): 4.35; 95% confidence interval (CI): 1.14-16.67); female sex * physical activity 4 or 5 times a week (OR: 7.86; 95% CI: 2.28-27.05); female sex * physical activity 3 times a week (OR: 7.32; 95% CI: 2.09-25.58); female sex * physical activity 1 or 2 times a week (OR: 14.57; 95% CI: 4.28-49.57); and female sex * no physical activity (OR: 24.17; 95% CI: 7.21-80.97).

**CONCLUSION:**

The lower the weekly frequency of physical activity during the period of social distancing was, the greater the chances of having stress levels were, especially for women.

## INTRODUCTION

The COVID-19 pandemic may be having many psychological impacts on people, at both an individual and a community level.^1^^,^^2^ The few studies published to date have shown that in the initial phase of the COVID-19 outbreak, more than half of the respondents rated its psychological impact as moderate to severe, and about a third reported having moderate to severe anxiety.^3^

In relation to the current pandemic scenario, both the Brazilian Ministry of Health and the World Health Organization recommend social distancing. However, it is known that long periods in isolation can increase behaviors that lead to physical inactivity, thereby contributing to the development of anxiety and depression.^4^^,^^5^^,^^6^

Thus, maintaining some frequency of physical activity in the home (aerobic activities, stretching and muscle and bone-strengthening exercises), during this period of social distancing, could help control stress levels, such as anxiety and depression.^7^ It has been suggested that higher levels of physical activity are associated with decreased risk of future anxiety disorders and depression.^8^^,^^9^ These results have been confirmed through recent meta-analyses, in which regular physical activity was seen to be a protective factor against depression.^9^^,^^10^

Based on the numerous health benefits that physical activity may provide,^11^^,^^12^^,^^13^ and considering the importance of maintaining physical activity during the COVID-19 pandemic, especially for controlling stress levels, such as anxiety and depression,^7^^,^^8^^,^^9^^,^^10^ it is necessary to expand knowledge about the role of the weekly frequency of physical activity.

## OBJECTIVE

The objective of the study was to ascertain the relationship between the weekly frequency of physical activity and the level of stress among Brazilian adults during social distancing due to the coronavirus (COVID-19), and the interaction of sex with this association.

## METHODS

### Ethical considerations

The present study followed the rules for research involving human beings of the Brazilian National Health Council. It was approved by the local university research ethics committee (CAAE: 30750220.1.0000.0102; date: June 9, 2020).

### Procedures

This was a cross-sectional, epidemiological and correlational study carried out using a non-probabilistic sample of Brazilian adults who were selected according to convenience. The sample was invited (from April to May 2020) to participate in the survey through dissemination in digital media (WhatsApp, Telegram, Facebook, Twitter and e-mails). Through these media, those interested in participating in the study had access to a questionnaire (available through Google Forms), named: “Questionnaire on habitual physical activity practices, eating habits and psychological factors during social distancing due to the new coronavirus (COVID-19)”.

A total of 2,347 adults were evaluated. Respondents who were under 18 years old (n = 38) and duplicate responses (n = 74) were excluded. Individuals who answered the questionnaires incorrectly were treated as sample loss (n = 235). Thus, the final sample included consisted of 2,000 Brazilian adults.

To verify the statistical power of the sample, an *a posteriori* sample calculation was performed considering a 95% confidence level (α = 0.05), an 80% power (β = 20) and a prevalence of physical activity of three times per week during social distancing of 21.1% (distribution of the sample itself). With these parameters, the sample of 2,000 subjects made it possible to identify prevalence ratios above 1.28 as a risk and below 0.75 as protection. These calculations were performed on the calculator G*Power version 3.1.9.4 (G*Power, Dusseldorf, Germany).

### Independent variable − physical activity (PA)

To assess physical activity at home (aerobic activities, stretching and muscle and bone-strengthening exercises) during social distancing, the following question was asked: “How often have you been practicing physical activity during social distancing?” The response options were: No exercise, 1 or 2 times a week, 3 times a week, 4 or 5 times a week or 6 or 7 times a week. The respondents were also asked at what time they performed this physical activity on a daily basis. However, the frequency of physical activity during the week was considered for analysis.

### Dependent variable – stress level

The respondents’ stress level was assessed using the K10 instrument.^14^ This instrument consists of 10 questions and assesses anxiety and depression over the last 30 days. The first nine questions all begin “During the past 30 days, how often did you feel...” and are completed with the following: “... exhausted for no good reason?”; “... nervous?”; “... so nervous that nothing could calm you down?”; “... hopeless?”; “... restless or agitated?”; “... so restless that you couldn’t stand still?”; “... depressed?”; “... so depressed that nothing could cheer you up?”; and “... worthless?”. Finally, the last question is “During the past 30 days, how often did you feel that everything was an effort?”.

The answer options were obtained using a Likert scale (never, a little, part of the time, most of the time and all the time). These answers to the 10 questions were summed to generate a final score, which could range from 10 to 50. These scores were used to estimate the level of anxiety or depression. Thus, the participants were classified as presenting low stress (scores of 10 to 15), moderate stress (16 to 21), high stress (22 to 29) or very high stress (30 to 50). For respondents with a total score greater than 22, there is a greater risk of having a mental disorder.^15^

### Sociodemographic and health factors

Sex was self-reported (“male” or “female”). Likewise, age was informed by the participants and was then grouped as: 18-29 years, 30-39 years, 40-49 years, 50-59 years or over 60 years. The body mass index (BMI) was calculated from self-reported weight and height in the questionnaire. It was obtained by dividing weight (kilograms) by height (meters) squared and was classified as specified by the World Health Organization (WHO): “underweight” (< 18.5 kg/m²); “normal weight” (18.5 to 24.9 kg/m²); “overweight” (25 to 29.9 kg/m²); or “obesity” (≥ 30 kg/m²).^16^ The respondents were also asked whether they had increased their food consumption during social distancing, with the following response options: no, sometimes or yes.

### Data analysis and statistical procedures

The data were described in terms of simple and relative frequencies, according to the stress level of the subjects. The chi-square test was used to compare the frequencies of factors at different levels of stress.

Possible associations between different levels of stress and sex, frequency of physical activities during social distancing, age groups, body mass index and increased food intake during social distancing were investigated through crude multinomial logistic regression (the proportionality of odds had previously been verified), with their respective 95% confidence intervals.

Subsequently, the interaction of sex was tested in the relationship between weekly frequency of physical activities and stress during social distancing (for example: sex * frequency of physical activity). For this, the interaction term was introduced into the models for multinomial logistic regression analysis (with 95% confidence intervals).

All the analyses were performed using the SPSS software, version 24.0 (Chicago, IL, United States), with a significance level set at P < 0.05.

## RESULTS

The final sample was 2,000 adults (59.6% women), with pre-dominance of the age group between 30 and 39 years (34.2%). Approximately half of the sample (49.3%) was classified as having normal weight, 42.2% reported not having increased their food consumption and 23.8% were practicing physical activities 4 to 5 times a week. Regarding stress, the low level was seen most frequently in the sample (35.9%) (Table 1). Higher levels of stress were associated with the following: females, in comparison with males (OR: 5.30; 95% CI: 3.59-7.82); low frequency of weekly physical activity, in comparison with high frequency of weekly physical activity (4 or 5 times: OR: 2.58; 95% CI: 1.23-5.41; 3 times: OR: 3.01; 95% CI: 1.44-6.28; once or twice: OR: 4.71; 95% CI: 2.27-9.75; and not done: OR: 9.73; 95% CI: 4.80-19.69); and the age groups of 30 to 39 years (OR: 5.99; 95% CI: 1.41-25.32) and 18 to 29 years (OR: 17.61; 95% CI: 4.19-73.94), in comparison with the age group of 60 years and over. Higher levels of stress were inversely associated with the following: overweight, in comparison to obesity (OR: 0.56; 95% CI: 0.35-0.90); and increased food intake: sometimes (OR: 0.49; 95% CI: 0.32-0.73) and none (OR: 0.24; 95% CI: 0.16-0.35), in comparison with increased food intake (Table 2).

**Table 1.** Characteristics of this sample of Brazilians according to their stress levels during the social distancing due to COVID-19 (n = 2,000)

	Stress level								P
	Low		Moderate		High		Very high		
	n	%	n	%	n	%	n	%	
**Sex**									
Male	335^a^	16.8	400^b^	20.0	290^c^	14.5	167^d^	8.3	< 0.001
Female	383^a^	19.1	275^b^	13.8	114^c^	5.7	36^d^	1.8	
**Weekly frequency of physical activities during social distancing**									
6 or 7 times	136^a^	6.8	72b	3.6	27^b^	1.4	10^b^	0.5	< 0.001
4 or 5 times	179^a.b^	8.9	182^b^	9.1	80^a^	4.0	34^a^	1.7	
3 times	167	8.3	135	6.8	82	4.1	37	1.8	
Once or twice	127^a^	6.3	151^a.b^	7.5	105^b^	5.3	44^a,b^	2.2	
Not practiced	109^a^	5.5	135^a^	6.8	110^b^	5.5	78^c^	3.9	
**Age group**									
60 years and over	51^a^	2.5	23^b^	1.1	7^b^	0.4	2^b^	0.1	< 0.001
50 to 59 years	92^a^	4.6	63^a.b^	3.1	23^b^	1.1	9^b^	0.4	
40 to 49 years	172^a^	8.6	137^a.b^	6.9	71^a.b^	3.5	28^b^	1.4	
30 to 39 years	251	12.6	230	11.5	144	7.2	59	2.9	
18 to 29 years	152^a^	7.6	222^b^	11.1	159^b^	8.0	105^c^	5.3	
**Body mass index**									
Obese	87	4.3	86	4.3	63	3.1	36	1.8	0.713
Overweight	266	13.3	254	12.7	127	6.3	62	3.1	
Normal	356	17.8	325	16.3	206	10.3	98	4.9	
Underweight	9	0.4	10	0.5	8	0.4	7	0.4	
**Increased food intake during social distancing**									
Yes	129^a^	6.5	189^b^	9.4	147^c^	7.3	82^c^	4.1	< 0.001
Sometimes	192	9.6	221	11.1	137	6.9	60	3.0	
No	397^a^	19.9	265^b^	13.3	120^c^	6.0	61^b. c^	3.0	

^a,b,c,d^differ significantly.

**Table 2.** Factors associated with stress levels among this sample of Brazilians during the social distancing due to COVID-19 (n = 2,000)

	Stress level								
	Moderate			High			Very high		
	OR	95% CI		OR	95% CI		OR	95% CI	
**Sex**									
Male	1	-	-	1	-	-	1	-	-
Female	1.66	1.34	2.05	2.90	2.23	3.77	5.30	3.59	7.82
**Frequency of physical activities during social distancing**									
6 or 7 times	1	-	-	1	-	-	1	-	-
4 or 5 times	1.92	1.35	2.73	2.25	1.37	3.67	2.58	1.23	5.41
3 times	1.52	1.06	2.19	2.47	1.51	4.03	3.01	1.44	6.28
Once or twice	2.24	1.55	3.25	4.16	2.55	6.77	4.71	2.27	9.75
Not practiced	2.33	1.59	3.42	5.08	3.11	8.30	9.73	4.80	19.69
**Age group**									
60 years and over	1	-	-	1	-	-	1	-	-
50 to 59 years	1.51	0.84	2.73	1.82	0.73	4.53	2.49	0.51	11.98
40 to 49 years	1.76	1.02	3.03	3.00	1.30	6.94	4.15	0.95	18.02
30 to 39 years	2.03	1.20	3.43	4.18	1.84	9.45	5.99	1.41	25.32
18 to 29 years	3.23	1.89	5.52	7.62	3.35	17.31	17.61	4.19	73.94
**Body mass index**									
Obese	1	-	-	1	-	-	1	-	-
Overweight	0.65	0.44	0.97	0.65	0.44	0.97	0.56	0.35	0.90
Normal	0.79	0.55	1.15	0.79	0.55	1.15	0.66	0.42	1.04
Underweight	1.22	0.44	3.35	1.22	0.44	3.35	1.88	0.65	5.43
**Increased food intake during social distancing**									
Yes	1	-	-	1	-	-	1	-	-
Sometimes	0.78	0.58	1.05	0.62	0.45	0.86	0.49	0.32	0.73
No	0.45	0.34	0.59	0.26	0.19	0.36	0.24	0.16	0.35

OR = odds ratio; CI = confidence interval; P < 0.05.

When sex was inserted as an interaction term in the association between the weekly frequency of physical activity and the stress level, significant values were presented for the following: male sex * no physical activity (OR: 4.35; 95% CI: 1.14-16.67); female sex * physical activity 4 or 5 times a week (OR: 7.86; 95% CI; 2.28-27.05); female sex * physical activity 3 times a week (OR: 7.32; 95% CI %: 2.09-25.58); female sex * physical activity 1 or 2 times a week (OR: 14.57; 95% CI: 4.28-49.57); and female sex * no physical activity (OR: 24.17; 95% CI: 7.21-80.97) (Table 3).

**Table 3.** Interaction of sex in the association between weekly frequency of physical activity and stress levels among this sample of Brazilians during the social distancing due to COVID-19 (n = 2,000)

	Stress level								
	Moderate			High			Very high		
	OR	95% CI		OR	95% CI		OR	95% CI	
Weekly frequency of physical activity * Male									
6 or 7 times	1	-	-	1	-	-	1	-	-
4 or 5 times	2.02	1.18	3.44	1.86	0.82	4.21	1.24	0.30	5.14
3 times	1.35	0.77	2.35	1.92	0.84	4.34	2.98	0.82	10.80
Once or twice	2.01	1.13	3.57	2.76	1.20	6.34	0.95	0.18	4.90
Not practiced	2.22	1.21	4.06	3.62	1.55	8.47	4.35	1.14	16.67
Weekly frequency of physical activity * Female									
6 or 7 times	1.48	0.82	2.65	1.77	0.74	4.23	2.07	0.51	8.35
4 or 5 times	2.93	1.70	5.04	4.95	2.27	10.82	7.86	2.28	27.05
3 times	2.76	1.58	4.81	5.83	2.66	12.78	7.32	2.09	25.58
Once or twice	3.71	2.13	6.46	9.36	4.31	20.30	14.57	4.28	49.57
Not practiced	3.51	2.01	6.13	10.07	4.65	21.80	24.17	7.21	80.97

OR = odds ratio; CI = confidence interval; *Interaction = multiplication of the possible moderating variable (sex) in the relationship between weekly frequency and stress during distancing; P < 0.05.

## DISCUSSION

Regarding the initial hypothesis of this study, the analyses confirmed the premise that the lower the weekly frequency of physical activity during the period of social distancing was, the greater the chances were that the subjects would present high levels of stress, even after considering adjustments for overweight and obesity,^17^ age and food consumption,^18^ which are factors that can also contribute to higher levels of stress. The Physical Activity Guidelines recommend that all adults, even those with chronic medical conditions, should engage in at least 150 minutes (recommended minimum) to 300 minutes a week of moderate-intensity exercise, if they are able to.^19^

These minimum recommendations are equivalent to something like 30 minutes a day of physical activity, 4 to 5 days a week, in order to obtain health benefits.^19^ The present study showed that adults (regardless of sex) who reported doing physical activity at this frequency in a regular week would be twice as likely to experience moderate stress and three times as likely to experience high and very high stress, compared with those who did higher frequencies of physical activities (6 to 7 times a week). These stress values (moderate, high and very high) increased as the frequency of physical activity in the week decreased. This explains not only the importance of complying with the minimum recommendations, but also the protective role that regular physical activity can play regarding health, when it is done at frequencies higher than the recommended minimum.^19^

Our results highlighted that individuals who had ceased to do physical activity showed significantly higher levels of stress. These findings were concordant with the results from previous studies in which the benefits of physical activity were investigated. Physical activity has a major role in mental health and cognitive function, because exercise has positive effects with regard to preventing and alleviating depression and anxiety.^10^^,^^20^^,^^21^ Our findings corroborate those of other studies that have investigated mental health and physical activity during COVID-19.^22^^,^^23^

For men, this association was not observed for high and very high stress, which would be in line with the recommendations, because with frequencies of physical activity of four to five times a week, the psychological responses seem to improve considerably.^10^^,^^20^^,^^21^^,^^22^^,^^23^ In contrast, if women were to follow the recommendations, this would not be enough to decrease the chances of moderate, high and very high stress. For women, practicing physical activities six or seven times a week did not show any association with different levels of stress. The fact is that, according to the literature, women are more prone to higher levels of stress.^10^^,^^20^^,^^21^^,^^22^^,^^23^ The results from the present survey indicate that, despite the lack of significant values, higher frequency of physical activities during the social distancing period would indicate a lower level of stress.

Despite the recommendations that home confinement (lockdown) should not prevent people from being physically active,^24^ the results from our study showed that a portion of the sample investigated did not do any physical activity during the COVID-19 home confinement period. It is likely elderly individuals have difficulty in exercising alone at home and have less access to remote physical activity areas. This may explain the reduction in physical activity among elderly people during this period.^23^^,^^24^ Further studies are needed to understand why women experience higher levels of stress than those of men.

In short, the present study contributes to enabling better understanding of the literature on the importance of maintaining a regular frequency of physical activity.^24^ For individuals who reduce their weekly frequency of physical activity, there is an extremely high chance of having high levels of stress, related to anxiety and depression.^14^

Some limitations of our study need to be considered in order to better understand the results. Reverse causality, which is a common feature in studies with a cross-sectional design, does not allow investigation of a cause-and-effect relationship or determination of the direction of the relationships. Nonetheless, this design has been used in several studies like ours. The use of reported measurements depends on the accuracy and recall power of the respondent’s responses. However, because our study was large, and because of the special conditions of distancing currently imposed in the vast majority of countries around the world, the use of questionnaires may be the best alternative. It was not possible to assess the type of physical activity (aerobic activities, stretching or muscle and bone-strengthening exercises), since the questionnaire spoke of physical activity in general. Future studies can ascertain the action of the type of physical activity performed.

## CONCLUSIONS

The lower the weekly frequency of physical activity during the period of social distancing was, the greater the chances were that the subjects would present high levels of stress. The interaction term (sex) showed that, especially for women, the reduction in training frequencies during the week contributed to increases in moderate, high and very high levels of stress, 14-fold for women who maintained physical activity only 1 or 2 times a week and 24-fold for those who did not do any weekly physical activity. In summary, our study presents relevant findings about the importance of maintaining the frequency of physical activity, either at home or away from home.
